# Deep learning model for tongue cancer diagnosis using endoscopic images

**DOI:** 10.1038/s41598-022-10287-9

**Published:** 2022-04-15

**Authors:** Jaesung Heo, June Hyuck Lim, Hye Ran Lee, Jeon Yeob Jang, Yoo Seob Shin, Dahee Kim, Jae Yol Lim, Young Min Park, Yoon Woo Koh, Soon-Hyun Ahn, Eun-Jae Chung, Doh Young Lee, Jungirl Seok, Chul-Ho Kim

**Affiliations:** 1 Department of Radiation Oncology, Ajou University School of Medicine, Suwon, Republic of Korea; 2 Department of Otolaryngology, Ajou University School of Medicine, 164 Worldcup-ro, Yeongtong-gu, Suwon, 16499 Republic of Korea; 3 Department of Otorhinolaryngology, Yonsei University, Seoul, Republic of Korea; 4 Department of Otorhinolaryngology-Head and Neck Surgery, Seoul National University Hospital, Seoul, Republic of Korea; 5 Department of Otorhinolaryngology-Head & Neck Surgery, National Cancer Center, Goyang, Republic of Korea

**Keywords:** Cancer, Cancer imaging, Cancer screening, Head and neck cancer, Translational research

## Abstract

In this study, we developed a deep learning model to identify patients with tongue cancer based on a validated dataset comprising oral endoscopic images. We retrospectively constructed a dataset of 12,400 verified endoscopic images from five university hospitals in South Korea, collected between 2010 and 2020 with the participation of otolaryngologists. To calculate the probability of malignancy using various convolutional neural network (CNN) architectures, several deep learning models were developed. Of the 12,400 total images, 5576 images related to the tongue were extracted. The CNN models showed a mean area under the receiver operating characteristic curve (AUROC) of 0.845 and a mean area under the precision-recall curve (AUPRC) of 0.892. The results indicate that the best model was DenseNet169 (AUROC 0.895 and AUPRC 0.918). The deep learning model, general physicians, and oncology specialists had sensitivities of 81.1%, 77.3%, and 91.7%; specificities of 86.8%, 75.0%, and 90.9%; and accuracies of 84.7%, 75.9%, and 91.2%, respectively. Meanwhile, fair agreement between the oncologist and the developed model was shown for cancer diagnosis (kappa value = 0.685). The deep learning model developed based on the verified endoscopic image dataset showed acceptable performance in tongue cancer diagnosis.

## Introduction

Oral cancer accounts for almost 3% of all cancer cases diagnosed worldwide^1^. According to the World Health Organization, more than 370,000 cases of oral cancer were reported in 2020^2^. Several studies have shown that tongue cancer is the most common type of oral cancer (42%)^3,4^. Oral cancer is prevalent in individuals mostly from Asia (65.8%) and is ranked one of Asia’s sixth most frequent malignancies^5^. The lifestyle of the Asian population, which includes such as chain-smoking, alcohol consumption, and betel quid chewing, is a strong risk factor for oral cancer^6,7^.

The early detection of tongue cancer is essential^8,9^. The overall 5-year survival rate for patients with tongue cancer is 68.1%^10^. According to the Surveillance, Epidemiology, and End Results database, the 5-year survival rates for local, regional, and distant stages are 82%, 68%, and 40%, respectively. In addition to the prognosis, patients with advanced tongue cancer experience difficulties during eating and speaking^11^. Furthermore, when the diagnosis is delayed, the scope of surgery broadens, and various invasive treatments are performed, resulting in increased side effects after treatment^12^.

Endoscopy is a simple, effective, and non-invasive method for diagnosing tongue cancer^13^. However, only a few specialists have the ability to accurately read endoscopic results. For example, if a suspicious lesion is identified in a local clinic, the patient should be referred to a specialist for confirmation of disease status and further management^14^. However, general physicians who lack experience in treating patients with tongue cancer might mistakenly diagnose visual patterns for signs of ulceration or oral mucosa disease^15^.

Studies on early detection of various malignancies using the characteristics of the tongue have been undertaken in the past^16–18^. Recently, the development of a primary diagnosis method through artificial intelligence (AI) analysis of oral endoscopic images can improve the chances of early diagnosis of tongue cancer. However, previous studies related to oral cancer were conducted with images created in non-clinical environments using smartphones or digital cameras, rather than in a validated medical environment by using an endoscope; further, the number of images was small (< 300 images)^19,20^. In addition, studies have shown that there is a risk that the existing diagnostic algorithms may misdiagnose or underestimate the risk to critically ill patients in clinical applications^21^. This result was attributed to the low quality of the data collected for AI learning^22^. Hence, in this study, we verified the quality of the constructed dataset. Based on this data, we explored the feasibility of endoscopy-imaging-based deep learning models for tongue cancer diagnosis.

## Results

### Dataset characteristics

We retrospectively constructed the dataset of 12,400 verified endoscopic images obtained from five university hospitals in South Korea between 2010 and 2020. Of the 12,400 total images, 5576 images related to the tongue were extracted. For the development and validation of the total dataset (N = 5576), 1941 endoscopic images of malignant lesions and 3635 non-malignant endoscopic images were included. A difference in the ratio between malignant and non-malignant tumors was confirmed by each medical institution (Table 1). The internal validation dataset contained 1809 photographs of malignant lesions and 3415 non-malignant lesions. The external validation dataset consisted of 132 photographs of malignant lesions and 220 non-malignant lesions.

**Table 1:** Dataset characteristics.

Hospital	Diagnosis	n	%
AUH	Non-malignancy	1867	75.71
	Malignancy	599	24.29
SNUH	Non-malignancy	157	23.54
	Malignancy	510	76.46
NCC	Non-malignancy	648	94.74
	Malignancy	36	5.26
BRH	Non-malignancy	220	62.50
	Malignancy	132	37.50
YUH	Non-malignancy	743	52.81
	Malignancy	664	47.19
Total	Non-malignancy	3635	65.19
	Malignancy	1941	34.81
	Total	5576	100.00

*AUH* Ajou University Hospital, *SNUH* Seoul National University Hospital, *NCC* National Cancer Center, *BRH* Boramae Medical Center, *YUH* Yonsei University Hospital.

### Parameter tuning and training

**Figure 1:**
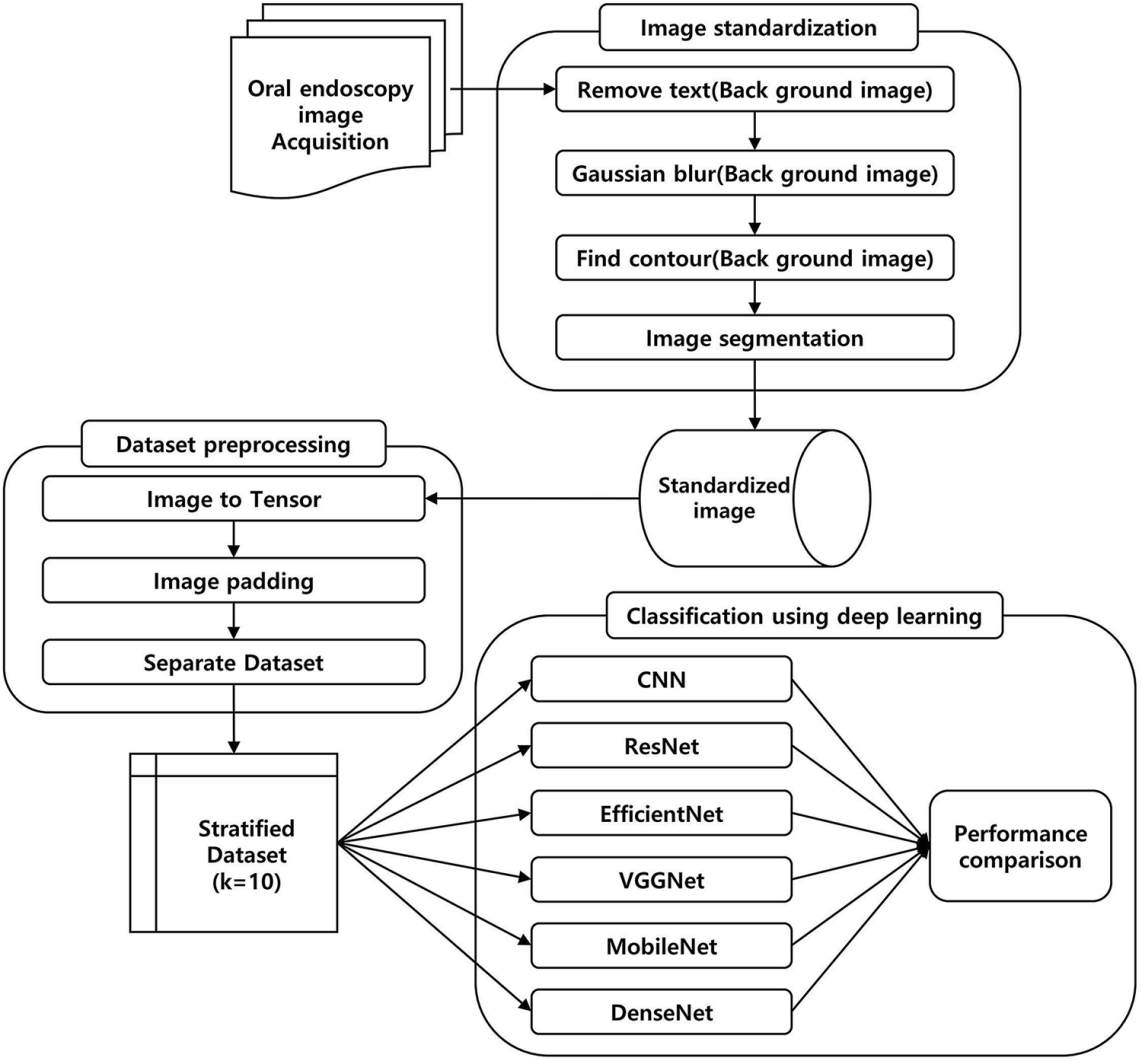
Overview of the development and evaluation of the tongue cancer diagnosis algorithm.

To perform fair comparison, all training hyperparameters were kept identical in all experiments (Fig. 1). The networks were trained for 300 epochs using binary cross-entropy loss with a batch size of 32. To avoid overfitting during training, we determined that overfitting occurred when the validation loss increased compared to the training loss, and then we explored ten additional epochs. If this trend continued, an early stopping logic that determines the parameter value in the epoch where the validation loss was increased compared to the train loss as the final parameter was applied. We did not use an algorithm that changes the learning rate according to the learning state, but rather applied Bayesian optimization to find the optimal learning rate to build the model.

### Testing and model selection

**Figure 2:**
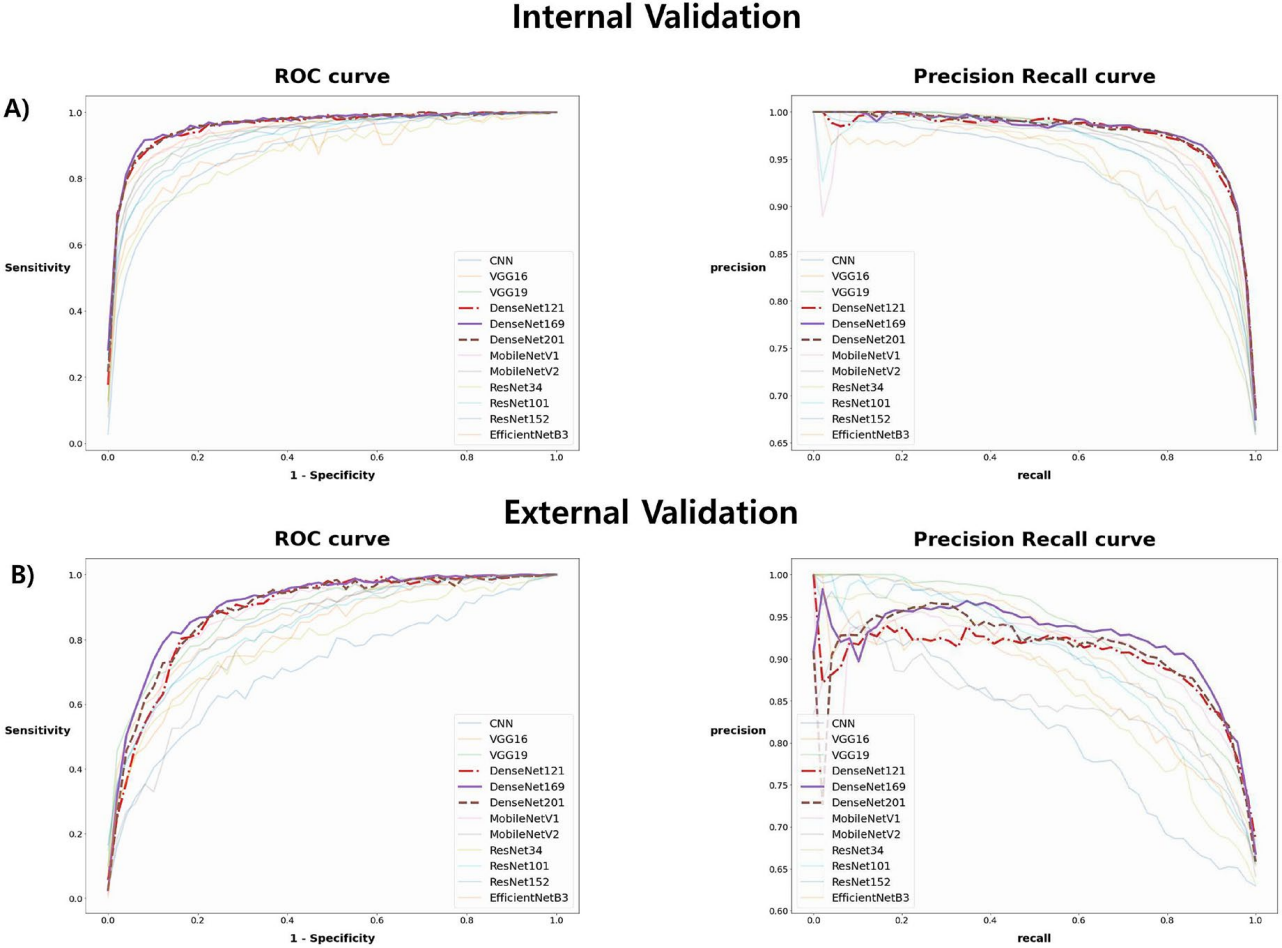
Receiver operating characteristic curves and precision-recall curves for the deep learning algorithm on internal validation dataset (**A**) and external validation datasets (**B**).

After training, we evaluated the classification models using internal and external validation datasets. The evaluation results are summarized in Table 2. The optimal point of the ROC curve was determined when the AUROC reached its maximum value. When AUPRC, AUROC, specificity, and F1-score were compared for different models, DenseNet models showed excellent performance. Among them, Densenet169 had a higher AUROC, AUPRC, and accuracy than DenseNet 201 and DenseNet 121. Therefore, DensetNet169 was selected as the final model (Fig. 2).

**Table 2:** Diagnostic performance of CNN models in internal validation (a) and external validation (b).

Model	Sensitivity (95% CI)	Specificity (95% CI)	Precision (95% CI)	F1-score (95% CI)	Accuracy (95% CI)	AUROC (95% CI)	AUPRC (95% CI)
**(a)**							
CNN	0.712 (0.685–0.739)	0.860 (0.839–0.881)	0.733 (0.706–0.760)	0.720 (0.693–0.747)	0.809 (0.785–0.833)	0.882 (0.862–0.902)	0.932 (0.917–0.947)
VGG16	0.822 (0.799–0.845)	0.911 (0.894–0.928)	0.832 (0.809–0.855)	0.826 (0.803–0.849)	0.880 (0.860–0.900)	0.950 (0.937–0.963)	0.974 (0.964–0.984)
VGG19	0.801 (0.777–0.825)	0.910 (0.893–0.927)	0.828 (0.805–0.851)	0.813 (0.789–0.837)	0.872 (0.852–0.892)	0.941 (0.927–0.955)	0.969 (0.959–0.979)
DenseNet121	0.886 (0.867–0.905)	0.913 (0.896–0.930)	0.844 (0.822–0.866)	0.864 (0.843–0.885)	0.904 (0.886–0.922)	0.959 (0.947–0.971)	0.977 (0.968–0.986)
DenseNet169	0.890 (0.871–0.909)	0.921 (0.905–0.937)	0.859 (0.838–0.880)	0.873 (0.853–0.893)	0.910 (0.893–0.927)	0.960 (0.948–0.972)	0.977 (0.968–0.986)
DenseNet201	0.866 (0.845–0.887)	0.928 (0.912–0.944)	0.866 (0.845–0.887)	0.865 (0.844–0.886)	0.907 (0.889–0.925)	0.960 (0.948–0.972)	0.978 (0.969–0.987)
MobileNetV1	0.817 (0.794–0.840)	0.913 (0.896–0.930)	0.840 (0.818–0.862)	0.822 (0.799–0.845)	0.879 (0.859–0.899)	0.946 (0.932–0.960)	0.969 (0.959–0.979)
MobileNetV2	0.612 (0.582–0.642)	0.925 (0.909–0.941)	0.819 (0.796–0.842)	0.782 (0.757–0.807)	0.817 (0.794–0.840)	0.931 (0.916–0.946)	0.961 (0.949–0.973)
ResNet34	0.690 (0.662–0.718)	0.842 (0.820–0.864)	0.709 (0.681–0.737)	0.687 (0.659–0.715)	0.789 (0.764–0.814)	0.873 (0.853–0.893)	0.934 (0.919–0.949)
ResNet101	0.710 (0.683–0.737)	0.905 (0.887–0.923)	0.802 (0.778–0.826)	0.749 (0.723–0.775)	0.838 (0.816–0.860)	0.920 (0.904–0.936)	0.957 (0.945–0.969)
ResNet152	0.744 (0.718–0.770)	0.908 (0.891–0.925)	0.812 (0.788–0.836)	0.775 (0.750–0.800)	0.851 (0.829–0.873)	0.926 (0.910–0.942)	0.960 (0.948–0.972)
EfficientNetB3	0.618 (0.589–0.647)	0.920 (0.904–0.936)	0.804 (0.780–0.828)	0.681 (0.653–0.709)	0.815 (0.791–0.839)	0.899 (0.881–0.917)	0.944 (0.930–0.958)
**(b)**							
CNN	0.767 (0.723–0.811)	0.563 (0.511–0.615)	0.521 (0.469–0.573)	0.614 (0.563–0.665)	0.639 (0.589–0.689)	0.716 (0.669–0.763)	0.818 (0.778–0.858)
VGG16	0.701 (0.653–0.749)	0.821 (0.781–0.861)	0.706 (0.658–0.754)	0.700 (0.652–0.748)	0.776 (0.732–0.82)	0.866 (0.830–0.902)	0.917 (0.888–0.946)
VGG19	0.642 (0.592–0.692)	0.893 (0.861–0.925)	0.784 (0.741–0.827)	0.704 (0.656–0.752)	0.799 (0.757–0.841)	0.887 (0.854–0.920)	0.930 (0.903–0.957)
DenseNet121	0.795 (0.753–0.837)	0.831 (0.792–0.870)	0.750 (0.705–0.795)	0.765 (0.721–0.809)	0.817 (0.777–0.857)	0.885 (0.852–0.918)	0.906 (0.876–0.936)
DenseNet169	0.793 (0.751–0.835)	0.853 (0.816–0.890)	0.773 (0.729–0.817)	0.777 (0.734–0.82)	0.830 (0.791–0.869)	0.895 (0.863–0.927)	0.918 (0.889–0.947)
DenseNet201	0.769 (0.725–0.813)	0.876 (0.842–0.910)	0.793 (0.751–0.835)	0.778 (0.735–0.821)	0.836 (0.797–0.875)	0.892 (0.860–0.924)	0.913 (0.884–0.942)
MobileNetV1	0.701 (0.653–0.749)	0.878 (0.844–0.912)	0.789 (0.746–0.832)	0.730 (0.684–0.776)	0.811 (0.77–0.852)	0.884 (0.851–0.917)	0.906 (0.876–0.936)
MobileNetV2	0.435 (0.383–0.487)	0.909 (0.879–0.939)	0.757 (0.712–0.802)	0.619 (0.568–0.67)	0.732 (0.686–0.778)	0.802 (0.760–0.844)	0.847 (0.809–0.885)
ResNet34	0.674 (0.625–0.723)	0.717 (0.670–0.764)	0.607 (0.556–0.658)	0.623 (0.572–0.674)	0.701 (0.653–0.749)	0.793 (0.751–0.835)	0.871 (0.836–0.906)
ResNet101	0.532 (0.480–0.584)	0.883 (0.849–0.917)	0.741 (0.695–0.787)	0.612 (0.561–0.663)	0.751 (0.706–0.796)	0.842 (0.804–0.880)	0.902 (0.871–0.933)
ResNet152	0.662 (0.613–0.711)	0.856 (0.819–0.893)	0.744 (0.698–0.79)	0.695 (0.647–0.743)	0.783 (0.740–0.826)	0.856 (0.819–0.893)	0.908 (0.878–0.938)
EfficientNetB3	0.524 (0.472–0.576)	0.865 (0.829–0.901)	0.739 (0.693–0.785)	0.572 (0.520–0.624)	0.737 (0.691–0.783)	0.816 (0.776–0.856)	0.873 (0.838–0.908)

### AI vs. human readers

**Figure 3:**
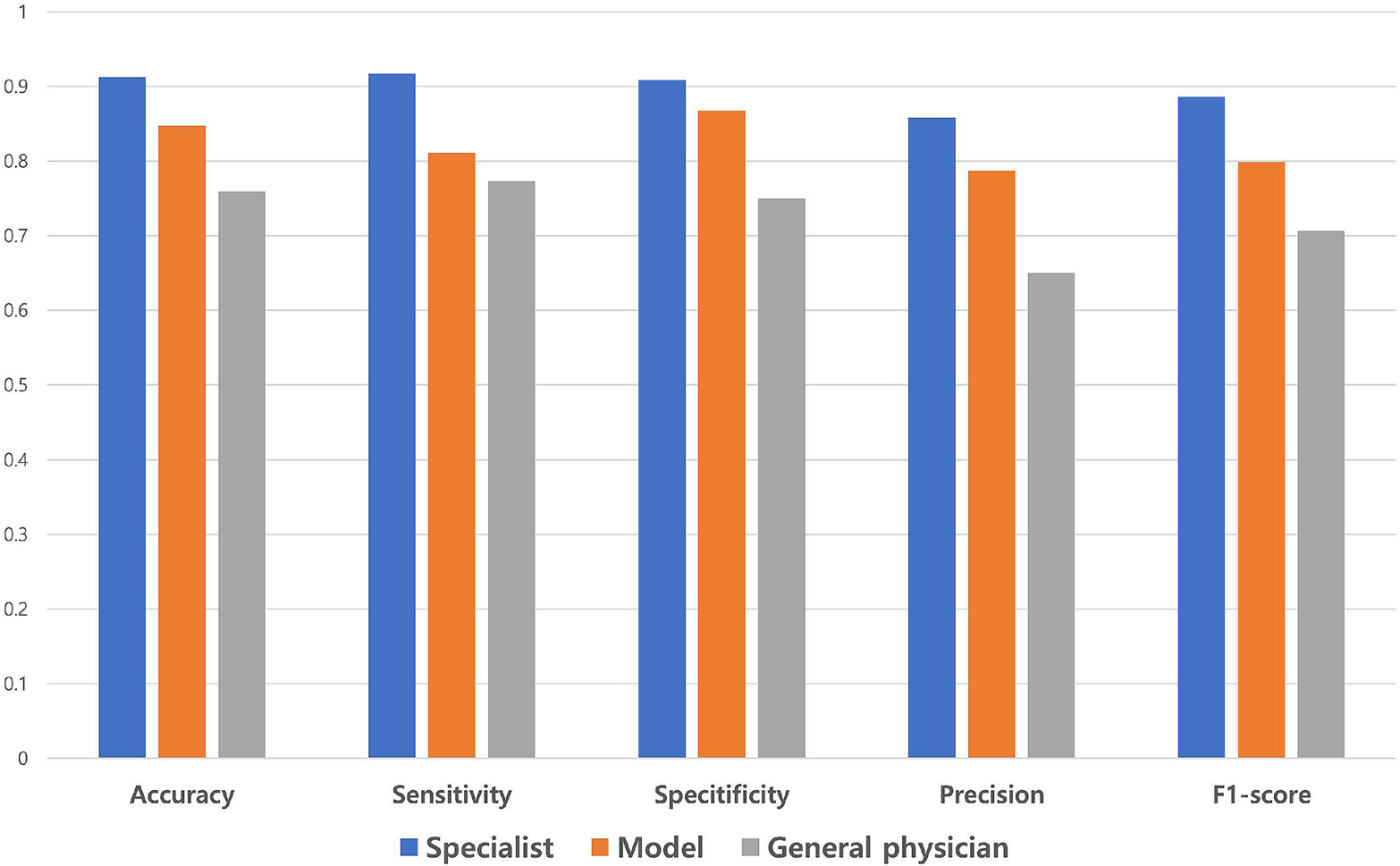
Performance of the deep learning model and comparison with human readers.

Figure 3 presents the test results for the best performing algorithm model and human readers on the external test dataset. The algorithm achieved an accuracy of 84.7% with a sensitivity of 81.1% and specificity of 86.8% for detecting tongue cancer. Among human readers, the accuracy of the oncology specialist was higher than that of the developed model at 92%. However, the accuracy of the general physician was lower than that of the model at 75.9%. The sensitivity and specificity considerably varied among the two human readers: the AI model achieved lower results than the specialist (sensitivity: 91.7%; specificity: 90.1%) and demonstrated significantly higher results than the general physician (sensitivity: 77.3%; specificity: 75.0%).

The agreement between the model and the human reader was estimated using the kappa value scale. Good agreement was observed between the model and the oncology specialist (kappa value = 0.685, 95% CI 0.606–0.763, p < 0.001). Further, moderate agreement was confirmed between the model and the general physician (kappa value = 0.482, 95% CI 0.389–0.575, p < 0.001). (Table 3).

**Table 3:** Agreement of the model and human readers.

	Malignancy prediction		
	Kappa value	95% CI	P value
**Model vs**			
Specialist	0.685	0.606–0.763	< 0.001
General physician	0.482	0.389–0.575	< 0.001

## Discussion

This study developed a deep learning algorithm based on DenseNet169 with acceptable performance (i.e., AUROC 0.895 and AUPRC 0.918 for external validation datasets) for tongue cancer diagnosis from endoscopic images (Table 2 and Fig. 2). Other existing medical imaging studies have yielded higher results in some cases. However, unlike this study, most of them have a limitation in that they showed internal validation results rather than performance validation results when using an external test set^23,24^. The AI model developed in our study could derive the visual patterns of cancer in cluttered oral endoscopic images. This AI-based diagnostic tool could have clinical significance for the early diagnosis of cancer.

Although the diagnosis of tongue cancer should be made early, it is sometimes delayed^25^. In this case, as the cancer stage increases, the prognosis worsens, and the scope of surgery expands, resulting in severe postoperative side effects, such as dysarthria^26^. Early detection is difficult, and from the patient’s viewpoint, knowledge and awareness regarding tongue cancer are lacking^27^. Furthermore, general physicians find it difficult to diagnose cancer in local areas using only endoscopic images^28^. Therefore, cancer should be diagnosed by an oncology specialist with extensive clinical experience. In previous studies, a screening system involving trained head and neck cancer specialists reduced oral cancer mortality^29^.

However, the number of specialists is small, and most of them work in large medical institutions, including university hospitals with low accessibility to patients. In the present study, the developed deep learning model had superior performance in diagnosing cancer than a general physician, but inferior than an oncologist (Fig. 3). The difference between these results is possible because general physicians have relatively little clinical experience with cancer patients^30^. This indicates that AI-based diagnosis models have the potential to help general physicians with little clinical experience in oncology treatment to diagnose endoscopic images. In other studies, examples of increased cancer diagnostic accuracy have been provided with the aid of AI^31^. In addition, when considering the results of the kappa coefficient, there was a good agreement between the model developed in this study and the specialist in terms of lesion classification (kappa value = 0.685, 95% CI 0.606–0.763) (Table 3). Therefore, as in gastrointestinal endoscopy, the developed model will enable general physicians to improve the accuracy of diagnosing tongue cancer by combining it with oral endoscopy that is available in primary medical institutions.

Recently, several studies have reported the usefulness of medical image analysis based on deep learning models. The CNN model based on ResNet-50 simultaneously learned to detect and characterize lesions on magnetic resonance imaging (MRI)^32^. In addition, the developed CNN model with VGGNet classified benign or malignant lesions in medial image data^33^. In this study, we retrained an existing CNN model developed on a large general natural image dataset using oral endoscopic images (Fig. 1). Six different models were used in this study: CNN, ResNet, EfficientNet, VGGNet, MobileNet, and DenseNet. Because the CNN model is the most basic model for image classification, it was used as a basis for comparing the performances of other models.

VGGNet, ResNet, and DenseNet were models that share a huge skeleton, and when the layers are deepened, each model can achieve better prediction performance. We were able to spot trends in the data and find an appropriate model using these associated models. MobileNet and VGGNet have relatively fast learning speeds, comply with the required performance, and are used to quickly check the results by adding logic to find data features more efficiently. ResNet, DenseNet, and EfficientNet are composed of deep layers; therefore, their learning speed is relatively slow, but their performance is acceptable. In particular, DenseNet shows superior performance with fewer parameters than ResNet. ResNet combines features by summation when passing through layers, but DenseNet is different because it concatenates the features rather than adding them.

Unlike previous studies that used standardized CT and MRI images, this study analyzed atypical oral images using the deep learning algorithm mentioned above. Since tongue cancer is a rare disease, we removed as much noise as possible from the image rather than increasing the amount of data. By minimizing the deviation of the data, the difference between the sample population and the overall population was reduced. DenseNet169, which was evaluated as the most suitable algorithm in this study, was also effective in image evaluation conducted in previous studies. In a study to classify pathological images in which atypical images were used similar to this study, effective results were obtained even with a small number of images^34^. Similarly, DenseNet169 showed the best performance in the study of the AI model for classifying the quality of tongue images^35^. Therefore, the application and optimization of AI algorithms considering the characteristics of each image data is essential. In particular, we believe that the model derived from this study will be meaningful for atypical data with large deviations among images, including endoscopes.

Despite recent innovative advances in deep learning technology, a large, validated dataset is one of the prerequisites for improving diagnostic performance. Driks emphasized the problem of “Frankenstein datasets”^22^. A Frankenstein dataset comprises information collected from multiple sources and assembled piece by piece. If an algorithm is tested with the same data used to train the model, it tends to appear to perform more accurately than it actually would on more realistic data or in practical applications. Therefore, we focused on well-organized and high-quality dataset construction. In the previous study, easily accessible smartphone and digital camera images were used; however, in this study, a dataset was constructed using oral endoscopy images created in clinical sites^19,20^. The poor-quality images could affect the analysis of image features and directly lead to a wrong diagnosis, causing severe interference with the development of the AI model. Therefore, oral endoscopic images are difficult to classify. In particular, oral endoscopy performed during the treatment process has different characteristics depending on the examiner because no guidelines were set for imaging.

This medical condition could lead to incorporation bias in the dataset. To create a relatively stable tongue image dataset, tongue images were collected using uniform endoscopic equipment. Additionally, to improve the quality of the dataset, several head and neck cancer specialists from multiple institutions directly participated in the data collection and review process. De-identification of data was carried out, and data inspection was performed more than twice. Moreover, a verification was conducted by TTA, an external institution. The radiomics approach used in previous studies involves manual ROI segmentation and extraction of several text features^36^. However, in this study, a deep learning network can be trained automatically without ROI segmentation. Therefore, advantages exist in terms of the decreased training time and costs for annotation workers. This method is designed to extract features directly from a dataset without the prerequisite for segmentation and manual processing. We performed processing to remove areas other than important areas so that the model could easily identify patterns in the image data.

We preprocessed the dataset before developing the AI model. The endoscopic images were of varying sizes, lighting conditions, and angles. In addition, owing to the noise of the equipment itself, some pixels sporadically entered as outliers in the oral endoscopy image. Some images also contained textual information, such as weather, and provided line guidelines (Fig. 1). In addition to the previous data preprocessing steps, such as scaling and adjusting the exposure, we developed a new algorithm and applied it in our research. For image standardization, we proceeded as follows. (1) We created a background image by converting the target image into a black-and-white image. (2) We removed the text from the background images. (3) We blurred the background image based on outliers using Gaussian blur. (4) The lesions were explored in the background image. (5) We cropped useless parts from the original image based on the lesions found in the background image (Supplement 1). All images were then converted into the JPEG format as required by our deep neural framework. According to the algorithms, they were then resized to 224 × 224 or 300 × 300 to the required input image size of the models before the model training process.

The current study had several limitations. First, the developed model cannot make a definite diagnosis for benign diseases among tongue lesions, such as leukoplakia and ulcers. In future studies, we plan to develop a model that can clearly distinguish benign and malignant tumors by classifying them into three categories: normal, benign, and malignant. Second, the oral endoscopic image characteristics used in this study differed from those of conventional CT and MRI images. These data have a high degree of freedom and are affected by the features of the endoscope user with atypical, non-standardized images. We used various data preprocessing techniques to compensate for these shortcomings. When collecting data in future studies, it would be beneficial to consider the application of endoscopy guidelines. Third, developing a cancer diagnosis model using only endoscopic images has a limit. In future research, high-performance diagnostic models are expected to be developed if images are combined with various clinical data. Fourth, several medical institutions participated in this study, resulting in differences between institutions in the amount of data, image characteristics, and the ratio of malignancy to non-malignancy (Table 1). In this study, data preprocessing was performed to correct this. In future research, uniformly distributing the ratio and amount of data for each participating institution would be necessary. Finally, lesions were not detected in this study. In future work, we plan to collect additional information on lesions and use it to develop an AI model that identifies suspected lesions with heat maps using Grad-CAM.

In conclusion, we have constructed a quality-validated dataset using oral endoscopy images from several medical institutions. A deep learning model based on the dataset showed acceptable performance for application in tongue cancer diagnosis. Compared with human readers, it showed lower diagnostic performance than oncology specialists and higher diagnostic performance than general physicians. Therefore, the developed algorithm could be used as an assistant tool for general physicians to increase the diagnosis and screening of cancer in clinical settings.

## Methods

### Dataset

We retrospectively collected 12,400 clinical endoscopic images from five hospitals in South Korea (i.e., Seoul National University Hospital, Yonsei University Hospital, Ajou University Hospital, National Cancer Center, and Boramae Medical Center) between December 9, 2010, and September 24, 2020. Through a database query of the medical databases (i.e., EMR and PACS), we extracted the endoscopic images taken for diagnosis of tongue cancer and the pathological reports of the images. The extracted endoscopic images were read and reviewed by at least two head and neck oncologists at each hospital, and image preprocessing, such as de-identification, was performed. The diagnosis results of each oral imaging image can be classified as malignant, benign, or normal. Among these, benign and normal images were classified as non-malignant images. The constructed dataset has undergone and passed an external verification by the Telecommunications Technology Association (TTA) for data structure and format accuracy.

**Figure 4:**
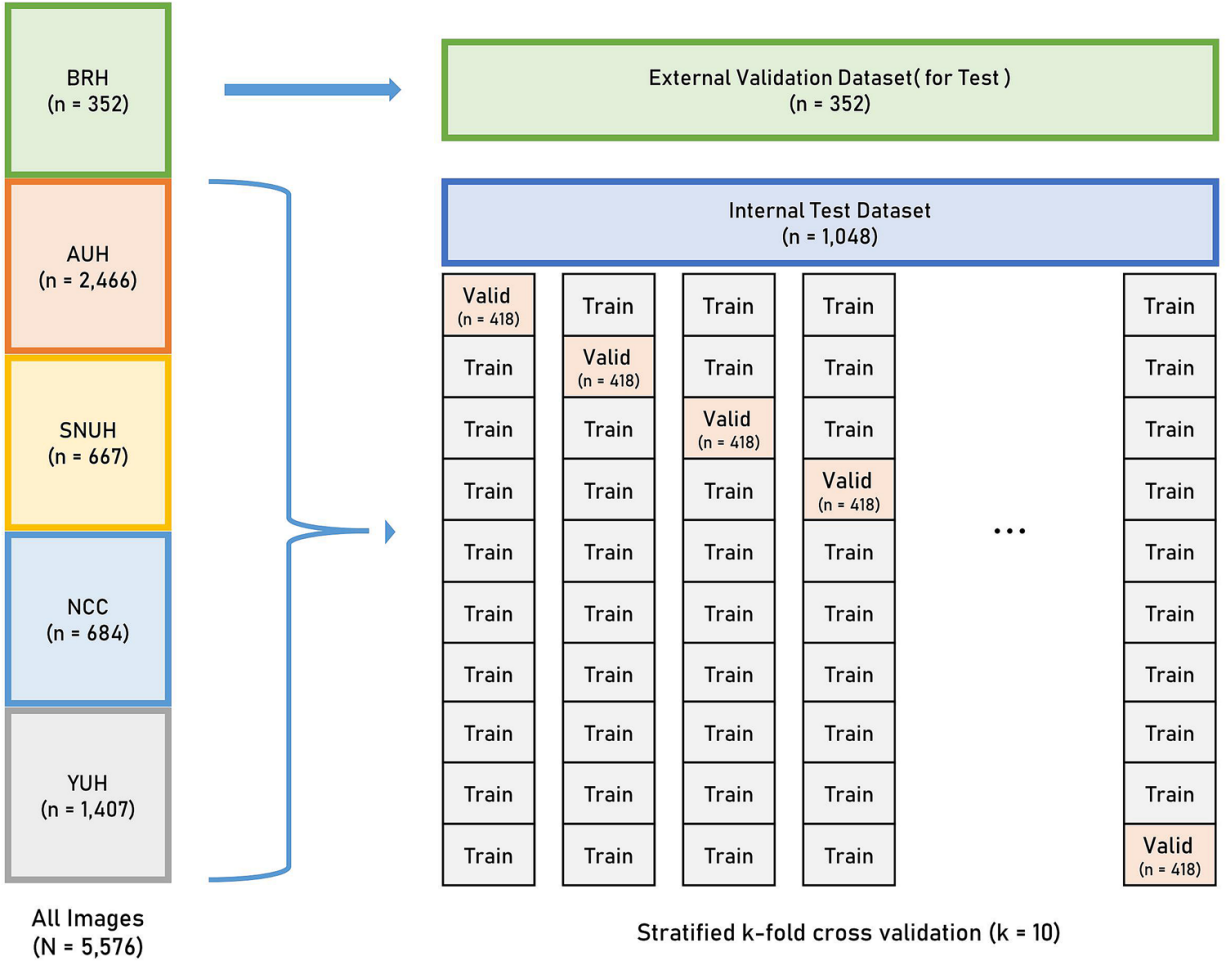
Validation and test structure diagram of the tongue cancer dataset for deep learning.

Of the 5576 total tongue images, we selected 5224 images (internal validation dataset) to develop the algorithm and then used the remaining 352 images (external validation dataset) for testing (Fig. 4). Pathological diagnosis was used as the correct answer to develop and validate the deep learning model. The Institutional Review Board of Ajou University Hospital approved this study (IRB No. AJIRB-MDB-20-311). Further, informed consent from all participants was waived by the IRB because of the retrospective nature of this study. All methods were performed in accordance with the Declaration of Helsinki.

### Deep learning model

To detect malignancy from oral endoscopic images (Fig. 1), we developed an automated deep learning algorithm using a cascaded convolutional neural network (CNN). The backbone networks for the detection and classification were initialized using a pre-trained model, which was trained with tens of millions of images in the ImageNet dataset and was further finetuned using the development dataset^37^. The tensor converted from the image was subjected to data scaling, data-type adjustment, and padding to maintain the image ratio. To optimize the hyperparameter, we used a Bayesian optimization method for the training and internal validation processes^38^. The target of Bayesian optimization was the area under a receiver operating characteristic curve (AUROC), and the hyperparameters that maximize AUROC were derived. The minibatch size was determined to be 32 to further improve the generalization performance. After the optimal hyperparameters were determined, we obtained the best model and evaluated its performance in the testing set.

A CNN architecture was constructed to calculate the probability of malignancy of an endoscopic image using ResNet (i.e., ResNet34, ResNet101, and ResNet152)^39^, EfficientNet B3^40^, VGGNet (i.e., VGG 16 and VGG 19)^41^, MobileNet (i.e., MobileNetV1 and MobileNetV2)^42^, and DenseNet (i.e., DenseNet121, DenseNet169, and DenseNet201)^43^. These models are neural networks with several layers and are commonly used for image classification. We applied stratified k-fold cross-validation to assess the deep learning model (k = 10). A total of 10 random datasets were extracted by fixing the seeds to ensure that the non-malignant and malignant ratios were equal. During internal validation, we randomly partitioned the dataset into approximately 70% training, 10% validation, and 20% test sets (Fig. 4). Moreover, we determined the number of epochs using an early termination tool. In this process, a dataset consisting of images obtained from Seoul National University Hospital, Severance Hospital, Ajou University Hospital, and National Cancer Center was used for internal validation. Through this method, the risk of overfitting increases from the moment the validation loss increases compared to the training loss. Thus, the training was ended after additional exploration.

After training the models, we examined the accuracy of the trained models by other clinical research centers in distinguishing non-malignant from malignant for external validation. To this end, we constructed a new testing dataset including 352 tongue images using the Boramae Medical Center dataset.

### Comparison with observer classification

We compared the performance of the algorithm with that of human readers using an external validation dataset. The human readers employed in our study were divided into two groups according to their professional backgrounds and clinical experiences. A specialist human reader who was a head and neck surgical oncologist with more than seven years of clinical experience participated in this study. The general physician human reader was a doctor with four years of experience after obtaining their license and was a non-specialist.

The human reader reviewed the same dataset and classified cases as malignant vs. non-malignant, without any prior knowledge on the patient history. The reader blindly evaluated the de-identified endoscopic image of the data and assessed the possibility of malignancy. The AI model with the best performance among the models was also evaluated using the same dataset.

The performance of the readers was assessed by comparing their predictions with the corresponding pathological reports. We evaluated the final results and calculated the overall accuracy, sensitivity, and specificity. We estimated the kappa values with linear weighting and 95% confidence intervals (CIs) to compare the diagnostic results of human readers and the model. The kappa value scale for agreement strength was as follows: poor: < 0.2; fair: 0.21–0.40; moderate: 0.41–0.60; good: 0.61–0.80; and very good: 0.81–1.00^44^.

### Statistical analysis

We evaluated the performance of the classification models using objective evaluation metrics, including specificity, precision, sensitivity, F1-score, and accuracy. The metrics base their mathematical foundation on the true positive (TP), true negative, false negative, and false-positive (FP) values of the models’ predictions. In addition, we used AUROC to evaluate the performance of the deep learning algorithm for distinguishing malignant from non-malignant. We plotted the receiver operating characteristic (ROC) curve by calculating the TP rate (sensitivity) and the FP rate (1 − specificity) with different predicted probability thresholds, and then we calculated the AUC values. Because the distribution of binary cases was not uniform, we also estimated the area under the precision-recall curve (AUPRC) values to evaluate the trained model. The corresponding 95% confidence interval was computed for each indicator value. The performance of the CNN models and the two readers in distinguishing malignant from non-malignant images was evaluated using these indicators.

We selected the model that best classified the endoscopic images by comparing the model performance. When selecting a model, the performance was evaluated by considering the first AUROC and the second AUPRC. Even if the model showed high performance in internal validation, the model that showed poor performance for external validation was excluded from model selection. All statistical analyses were performed using pandas (version 0.22.1), scikit-learn (version 0.24.1), NumPy (1.19.5), Matplotlib (3.3.4), OpenCV-Python (4.5.2), and Bayesian optimization (1.2.0) Python packages. We used Keras, which is a deep learning framework that acts as an interface for the TensorFlow2 library. Model structures were developed on graphical processing unit servers with multiple NVIDIA Tesla V100 graphic process units (32 GB × 4) and Xeon Gold 6248 (2.5 GHZ/20-core/150 W, 512 GB RAM) as the central processing unit.

### Ethical statement

The Institutional Review Board of Ajou University Hospital approved this study (IRB No. AJIRB-MDB-20–311). Further, informed consent from all participants was waived by the IRB because of the retrospective nature of this study.

## Supplementary Information

Supplementary Legend.

Supplementary Figure.

## Data Availability

The datasets generated and/or analyzed in this study are available from the corresponding author upon reasonable request.

## Code availability

To train the classification model in this study, we used the publicly available TensorFlow training script available at https://github.com/tensorflow/models/tree/master/official/vision/image_classification.
