# Supplementary material for: Deep learning model for tongue cancer diagnosis using endoscopic images
Source: Sci Rep. 2022 Apr 15;12:6281. doi: 10.1038/s41598-022-10287-9 (PMC9012779; doi:10.1038/s41598-022-10287-9)
Supplement: Supplementary file 1 — Supplementary Legend. [file 41598_2022_10287_MOESM1_ESM.docx]

**Supplement 1**. Data preprocessing process through image standardization
