# Supplementary figures and images for: Deep learning model for tongue cancer diagnosis using endoscopic images
Source: Sci Rep. 2022 Apr 15;12:6281. doi: 10.1038/s41598-022-10287-9 (PMC9012779; doi:10.1038/s41598-022-10287-9)

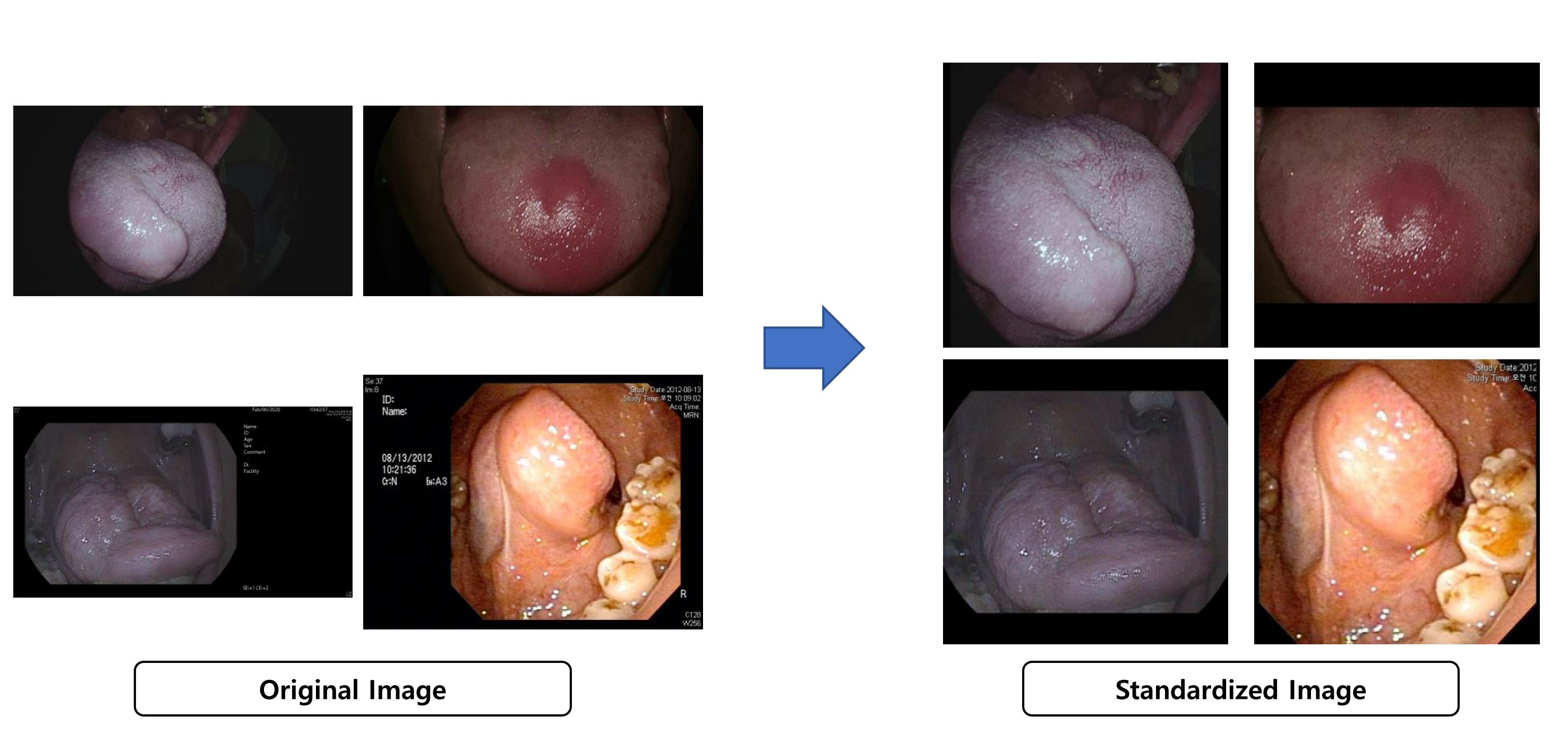

Supplement: Supplementary file 2 — Supplementary Figure. [file 41598_2022_10287_MOESM2_ESM.jpg]
